# Quantitative Investigation of Acoustic Emission Waveform Parameters from Crack Opening in a Rail Section Using Clustering Algorithms and Advanced Signal Processing

**DOI:** 10.3390/s22228643

**Published:** 2022-11-09

**Authors:** Harsh Mahajan, Sauvik Banerjee

**Affiliations:** Department of Civil Engineering, Indian Institute of Technology Bombay, Mumbai 400076, India

**Keywords:** acoustic emission testing, rail monitoring, unsupervised learning, bending experiment

## Abstract

Acoustic emission (AE) is an emerging technology for real-time non-destructive testing of structures. While research on a simulated AE source in rail and testing on rail material using small beam samples have been conducted, a study is required in lab environment to investigate AE waveform characteristics generated by crack in rail. In this paper, a three-point bending test is conducted on an actual rail section of 1500 mm with transverse damage of 38% head area to simulate AE source due to crack opening in the rail. AE signals are recorded for three different loads. For data analysis, unsupervised machine learning algorithms such as k-means, fuzzy-C mean and gaussian mixture model are used to cluster and filter out usable signals from the whole dataset corrupted by noisy signals from various sources. k-mean with principal component was observed to be best technique based on silhouette score. The frequency and amplitude of waveform have been discussed in relation to load and crack opening displacement. This study establishes a baseline for linking load, crack opening, and AE wave characteristics. This work can ultimately aid in the development of robust denoising, and damage analysis algorithms based on the frequency content and dispersion of the AE waveform.

## 1. Introduction

**Figure 1:**
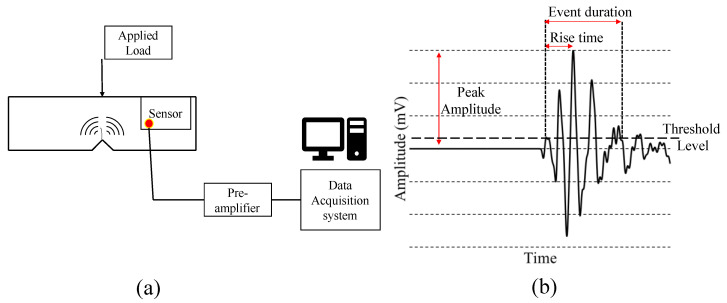
AE testing of structure (**a**) AE testing setup (**b**) Typical AE signal.

Railways play a crucial part in the country’s economy as a faster and more efficient mode of transportation. However, rail service condition is limited by fatigue caused by dynamic loads, chemical attacks, and manufacturing flaws. The health monitoring of rail section is essential to avoid derailment which may result in direct and indirect cost. In most cases, conventional ultrasonic testing is preferred for rail section monitoring. However, new techniques must be developed due to the limitations of ultrasonic testing in terms of testing speed and cascading of significant damage due to minor surface flaws. Acoustic emission is a rapidly evolving non-destructive testing method for inspecting concrete, metallic, and composite structures. Acoustic emission is a wide band frequency ultrasonic wave produced when energy is released suddenly from damage under load. It is usually referred to as passive monitoring because it does not require an artificially generated source, enabling continuous condition monitoring of rail lines without interfering with train traffic. Figure 1 shows the basic concept of AE testing and typical AE signal.

Over the last two decades, acoustic emission testing of rail has been explored. Numerous researchers have attempted to examine the AE wave and establish a correlation between wave parameters and damage.

Bruzelius and Mba [1] pioneered the use of AE testing in the rail. The authors discussed the application and issues associated with rail acoustic emission testing. In the research conducted by Bollas et al. [2], a correlation between defect size and AE hits were detected. Damage-induced AE signals are usually corrupted by noise from various sources. Investigations have been undertaken to distinguish genuine AE signals from noisy data. Hao et al. [3] investigated the denoising approach based on wavelet transform. Liang et al. [4] proposed an approach for detecting rail surface defects that combines adaptive noise cancelling and time-frequency signal processing. The authors conducted an experiment using a roller test rig with damaged rail surfaces and determined that the suggested approach for denoising was effective. Li et al. [5] proposed a methodology for sizing cracks in rail steel by conducting a fatigue test on small notched rectangular test specimen derived from the rail head. The authors analysed the AE waveform generated during the fatigue cycle and correlated AE counts with crack size in a small sample.

Machine learning tools are also being utilized rapidly for damage parameter detection in complex geometries and various materials. Acoustic emission testing generates an enormous amount of data, and usable AE signal needs to be segregated from noisy data. Unsupervised ML algorithms can help cluster the AE signal based on various wave parameters [6,7]. Tang et al. [8] utilised feature selection based on k-mean clustering to have classification of AE failure mode in composite material of wind turbine blade. Pashmforoush et al. [9] employed k-means genetic algorithm to discriminate AE signal generated by mode I delamination in sandwich composite from different failure modes. Satour et al. [10] had utilized wavelet coefficient and k-means clustering for analysis of acoustic data. Steen and Vestrynge [11] developed a hierarchical clustering algorithm to distinguish AE source during corrosion process. ML application in various non-destructive testing has also rapidly increased, resulting in a more suitable damage detection algorithm [12,13,14,15,16].

**Figure 2:**
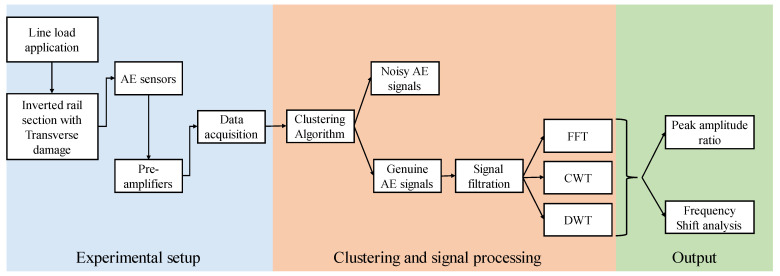
Technical flowchart of presented analysis on AE testing of rail.

The literature as described above addressed two distinct broad segments: AE testing of rail and machine learning algorithm application in non-destructive testing. However, the AE signal caused by the crack opening in the actual rail section is not adequately examined. The existence of multiple noise sources, the wide frequency range of the AE signal, and the complex structure of the rail all provide challenges when performing AE testing on rail sections. Bending test of simple notched beam section cannot duplicate actual AE signal in rail as its dispersion characteristics are quite different. Thus, research on a damaged rail in a laboratory environment is vital to generate AE signal parameters that may be utilised to guide subsequent investigations. In this research, a three-point bending test is performed on an inverted rail segment with a crack to simulate crack opening. The AE signals generated due to crack opening are collected and analysed. The clustering approach is also utilised to segregate genuine AE signals from the noisy dataset. Following that, a detailed investigation of the waveform parameters is done to acquire insights into the AE signal in the rail segment using fast Fourier transform (FFT), continuous wavelet transforms (CWT), and discrete wavelet transforms (DWT). This study contributes toward developing a real-time AE-based health monitoring system for rails. Clustering techniques based on waveform parameters can help to segregate genuine signals and to reduce the manual effort of extracting signals from a dataset containing various noise sources. AE waveform analysis of signals generated due to crack opening in rail section indicates the change in waveform parameters with respect to increasing load. The obtained information about characteristics of AE signals generated due to crack opening can help in more robust denoising and analysis techniques making AE testing of rail faster and more efficient. Figure 2 shows the technical flowchart of presented work.

## 2. Experimental Setup

**Figure 3:**
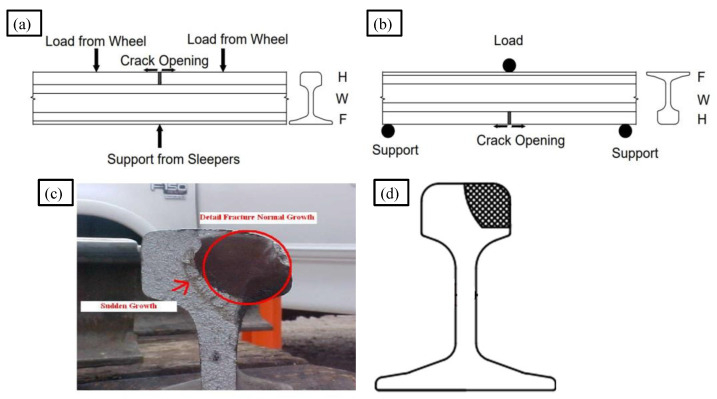
Concept behind three-point bending experiment of acoustic emission generated by crack opening in rail section (**a**) Crack under train load (**b**) Load on inverted rail (**c**) Actual damage in rail (**d**) Simulated damage.

A train traversing over a rail track imposes dynamic loading on the rail surface with variable amplitudes. The train movement generates an alternating cycle of tensile and compressive stress, thereby causing the damage in the rail section to suddenly open and close simultaneously. Opening of damage in rail results in the development of acoustic emission waves caused due to the formation of a plastic zone at the crack tip [17] and subsequent propagation of crack subjected to the fatigue cycle. One can better understand the damage severity by recording and analysing such signals. However, data collected during actual train testing contains a great deal of noise and can mislead the results if not filtered and appropriately analysed. Hence, to better understand the behaviour of the acoustic emission signals generated during loading, a three-point bending experiment on an inverted rail with transverse damage in the rail head was conducted in the laboratory. Laboratory experiment eliminates a few noise sources helping in proper analysis of AE signal. This experiment is being used to simulate the actual opening of a crack under stress and to generate AE waves associated to crack. Observing the various aspects of the AE wave created by the crack can aid in developing robust algorithms for identifying the AE source in field tests. EN 14730-1:2006 [18] was used as a guide for conducting the test. The test specimen is a 1500 mm long rail piece with 38% head area transverse damage in the centre. Figure 3 represents the insight behind the proposed laboratory experiment and transverse damage in rail. Transverse damage starts with minor cracks, usually on gage side of rail, which is in direct contact with the rail wheel flange. The crack gradually grows over the period and after attaining larger area, it propagates rapidly followed by rail failure.

**Figure 4:**
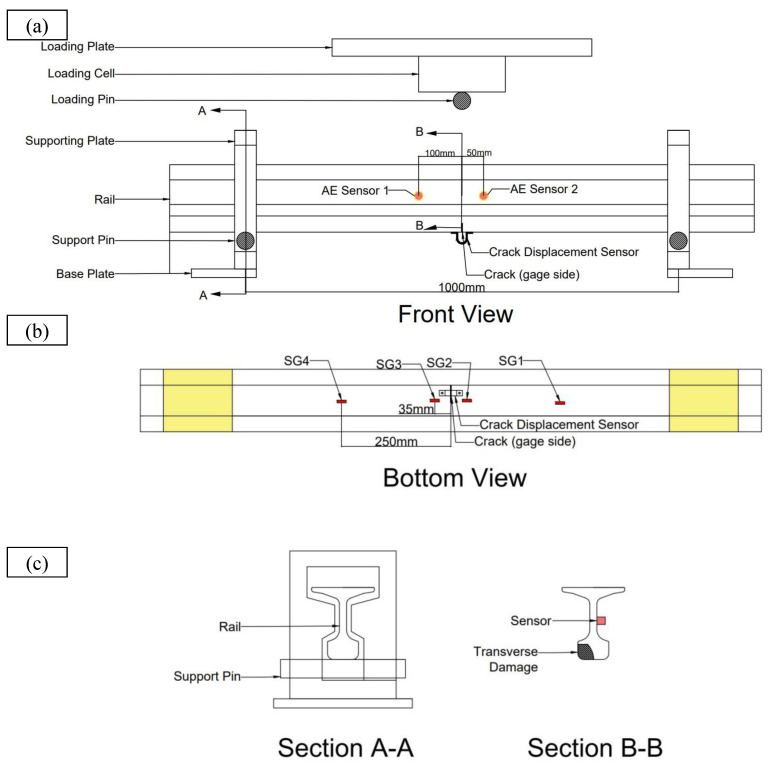
Schematics for experimental setup (**a**) front view (**b**) Position of strain gauge, crack and crack displacement sensor (**c**) End support and position of AE sensor.

**Figure 5:**
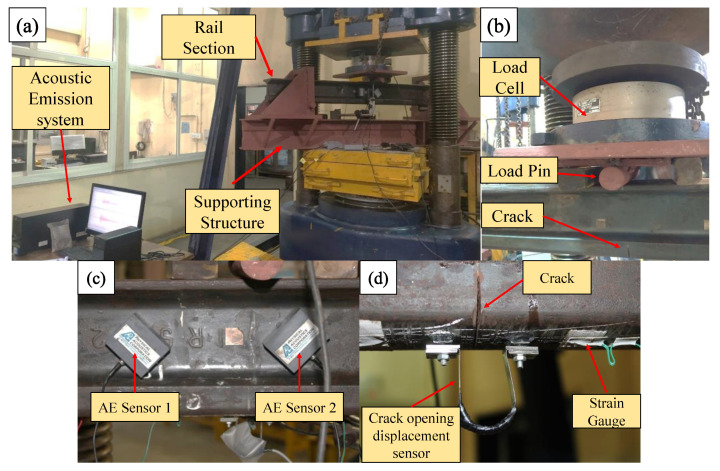
Pictures for actual experimental setup (**a**) Three-point bending setup and acoustic emission system (**b**) Loading arrangement (**c**) Acoustic emission sensor (**d**) Crack and crack opening displacement sensor.

Figure 4 illustrates the detailed schematics for the inverted rail bending test. Due to the instability of the inverted rail in the lateral direction, a substantial support structure was fabricated for safety. A transducer measuring crack opening displacement was mounted across the crack over the rail’s head part. Four strain gauges were mounted over the head on both sides of the crack. The Physical Acoustic Emission Corporation’s (PAC) Micro-II express digital AE equipment was utilised to measure and record AE signals during the bending test. PAC WS-α sensors with frequency band on 100–1000 kHz were coupled to the rail surface using magnetic couplers. To extract a less noisy signal, a threshold value of 55 dB was adopted. For the tests, the pre-amplifier gain was set at 40 dB. The specimen was loaded using a compressive loading machine equipped with a bending load arrangement. The AE hit is determined by hit definition time (HDT), hit lockout time (HLT), and peak definition time (PDT) [19]. For the case of metal, as per PAC AE manual [20], determination parameters are set as 300, 600 and 1000 µs for HDT, PDT, HLT respectively. The determination parameters are selected to avoid unnecessary noise while recording the AE signals. Three different loadings were applied on the specimen: 100 kN, 150 kN, and 200 kN. Loading was selected to avoid instability during testing of inverted rail section. The specimen was unloaded before every cycle. Vertical displacement was measured using a linear variable differential transformer (LVDT). Figure 5 illustrates the specifics of actual experimental setups.

Typically, research on the application of AE testing on rail has been conducted in the field. Nonetheless, the presence of noise sources on the field makes it challenging to distinguish the characteristics of AE signals generated by a rail crack. In addition, few studies have utilised tensile and bending tests on small samples of rail material. In this case, studies are limited by a mismatch between the dispersion characteristics of the rail section and the simple rectangular section. In the presented experiment method, multiple noise sources can be controlled, and different damage severity and sensor configurations can be tested. Thus, the presented AE testing method can aid in developing a full-fledged AE-based system for rail section health monitoring.

## 3. Clustering Algorithm and Signal Processing

### 3.1. Clustering Algorithm

Acoustic emission testing generated a huge amount of data due to various noise sources. It is essential to segregate the genuine signals from other sources in the dataset. To approach clustering, three clustering algorithms, namely, k-means [21], Fuzzy C-means and gaussian mixture model (GMM) [22] were used. Algorithms 1–3 hows the algorithm for k-mean, fuzzy C-means and gaussian mixture model for clustering respectively. The features that are extracted from waveforms are presented in Table 1. The features are wave-based, parameters consist of time domain and frequency domain characteristics of the waveform. These features might be correlated to each other. Hence, the principal component analysis (PCA) method was also utilised for dimension reduction, resulting in better performance and efficiency. PCA decreases the dimension of a dataset by projecting the data onto a subspace with lower dimensionality [23]. Using linear transformation, the algorithm transforms a p-dimensional data vector X into a q-dimensional data vector Z. Given the data X_i_ = (x_1i_, x_2i_,…, x_pi_) with i = 1,…, N, the new data vector is Zi = (z_1i_, z_2i_,…, z_qi_), where z_1_ is the linear combination of the original x_j_ (j = 1,…, p) with maximal variance, z_2_ is the linear combination, which explains the majority of the remaining variance, and so on, i.e., the This strategy is based on extracting a predetermined cumulative proportion of total variation from successive components. The objective was to assure the practical relevance of the generated variables by ensuring they explain a minimum amount of variance.
**Algorithm 1:** K-mean clustering**Input**X = {x_1_, x_2_, …………………, n_1_} // set of n data items K // number of desired clusters**Output**A set of K clusters**Steps**1.Initialize cluster centroids µ_1_, µ_2_, ……… µ_k_
$\in {\mathbb{R}}^{n}$ randomly
2.Repeat until convergence: {For every i, set $\;\;\;\;\;c^{\left(i\right)}={\mathrm{argmin}}_{j}\|x^{i}-\mu_{j}\|{}^{2}$ For every k, set $\;\;\;\;\;\;\mu_{j}=\frac{\sum_{i=1}^{m}1\;\left\{c^{\left(i\right)}=j\right\}x^{\left(i\right)}}{\sum_{i=1}^{m}1\;\left\{c^{\left(i\right)}=j\right\}}$ }

**Algorithm 2:** Fuzzy C-mean clustering
**Input**
X = {x_1_, x_2_, …………………, x_n_} // set of n data items c = number of desired clusters v_j_ = Centre of Cluster m = degree of fuzziness $\in \left(1,+\infty \right)$ T = Maximum number of iterations u_ij_ = Membership degree of the i^th^ datum in the j^th^ cluster i = 1,2,…..,n j = 1,2,……,c U = Fuzzy c-classified matrix of finite set V = Collection of X cluster centres
**Output**
A set of K clusters
**Steps**
1.Initialization of the c m, T and random initialisation of u_ij_
2.Determine cluster centre v_j_
$\;\;\;\;\;\;v_{j}=\frac{\sum_{i=1}^{n}u_{\mathrm{ij}}^{m}x_{i}}{\sum_{i=1}^{n}u_{\mathrm{ij}}^{m}}$
3.Determine the change in the membership function matrix $\;\;\;\;\;u_{\mathrm{ij}}=\frac{1}{\sum_{k=1}^{c}{\left[\frac{d_{\mathrm{ij}}}{d_{\mathrm{ik}}}\right]}^{\frac{2}{m-1}}}$ where, $\;\;\;\;\;\;d_{\mathrm{ij}}=\|x_{i}-v_{j}\|$
4.Calculate J(U,V) Membership and cluster centers are updated after each iteration by repeating steps 2 and 3 until the minimum ‘J’ value is achieved or $\|V^{\left(T+1\right)}-V^{\left(T\right)}\|<\beta$. where β is the termination criterion between [0,1] J is the objective function $\;\;\;\;J_{m}=\overset{n}{\underset{i=1}{\sum }}\overset{c}{\underset{j=1}{\sum }}u_{\mathrm{ij}}^{m}\|x_{i}-v_{j}\|{}^{2}$

**Algorithm 3:** Gaussian mixture modelling EM algorithmInputX= [x_1_, x_2_, …………………, x_n_] K= number of clusterOutputp(z_k_ =1|x_n_), values of $\mu_{k},\Sigma_{k},\pi_{k}$ for which objective log likelihood is minimumSteps1.Initialisation with $\mu_{k},\Sigma_{k},\pi_{k}$ and evaluate log likelihood $\;\;\;\ln p(X|\mu ,\Sigma ,\pi )=\overset{n}{\underset{i=1}{\sum }}\ln\left\{\overset{K}{\underset{k=1}{\sum }}\pi_{k}Ν(x_{i}|m_{k},\Sigma_{k})\right\}$
2.E-step $\;\;\;\;\;p\left(z_{k}=1|x_{i}\right)=\frac{\pi_{k}Ν\left(x_{i}|m_{k},\Sigma_{k}\right)}{\sum_{j=1}^{K}\pi_{j}Ν\left(x_{n}|m_{j},\Sigma_{j}\right)}$
3.M-step $\;\;\;\;\;\;\;\mu_{k}=\frac{1}{N_{k}}\overset{n}{\underset{i=1}{\sum }}p(z_{k}=1|x_{i})x_{i}$ $\;\;\;\Sigma_{k}=\frac{1}{N_{k}}\overset{n}{\underset{i=1}{\sum }}p(z_{k}=1|x_{i})\left(x_{i}-\mu_{k}\right){\left(x_{i}-\mu_{k}\right)}^{T}$
$\;\;\;\;\;\;\;\;\;\pi_{k}=\frac{N_{k}}{n}$ where, $\;\;\;\;\;\;N_{k}=\overset{n}{\underset{i=1}{\sum }}p(z_{k}=1|x_{i})x_{i}$
4.Calculate log likelihood with new set of data $\;\;\;\ln p(X|\mu ,\Sigma ,\pi )=\overset{n}{\underset{i=1}{\sum }}\ln\left\{\overset{K}{\underset{k=1}{\sum }}\pi_{k}Ν(x_{i}|m_{k},\Sigma_{k})\right\}$
5.Repeat step 2 to 4 until converged

### 3.2. Signal Processing

#### 3.2.1. Signal Filtering

Filtering of a signal is needed to isolate helpful information from the raw signal by eliminating unwanted components. Through digital signal processing, digital filters of various types can be utilised for such tasks. In this study, an infinite impulse response (IIR) filter has been considered since for a given filter order, it exhibits a much sharper transition required for the proper filtering of noise from the acquired AE signal [24]. In the IIR digital filter, the Elliptic filter was used to achieve a sharper cut off than the Chebyshev filter. The bandpass frequency range was kept between 125 kHz to 650 kHz based on the range of operating frequency and calibration curve of sensors. Filter parameters are provided in Table 2.

#### 3.2.2. Wavelet Transform

A wavelet transform represents the signal in frequency and time domain using dilation and transition parameters. Continuous wavelets transform (CWT) of the signal was done using a mother wavelet resulting in wavelet coefficients for different time and pseudo-frequencies. The value of wavelet coefficient and its association with time and frequency is an essential feature of a signal. The equation of CWT is given by Equation (1):

(1) $$\mathrm{CWT}\left(a,b\right)=\left(f,\psi_{a,b}\right)=\frac{1}{\sqrt{a}}\int_{-\infty }^{\infty }f\left(t\right)\psi^{*}\left(\frac{t-b}{a}\right)\mathrm{dt}$$

In CWT, calculating wavelet coefficients at every possible scale involves much work and huge data generation. If scales(a) and positions(b) are discrete, then the analysis will be much easier and will not generate massive data. For a given function f(k), the inner product 〈f,ψ_(m,n) 〉 then gives the discrete wavelet transform (DWT), which is given in Equation (2)

(2) $$\mathrm{DWT}\left(m,n\right)=a_{o}^{-\frac{m}{2}}\overset{\infty }{\underset{k=-\infty }{\sum }}f\left(k\right).\psi^{*}\left(a_{o}^{-m}k-{\mathrm{nb}}_{o}\right)$$

**Figure 6:**
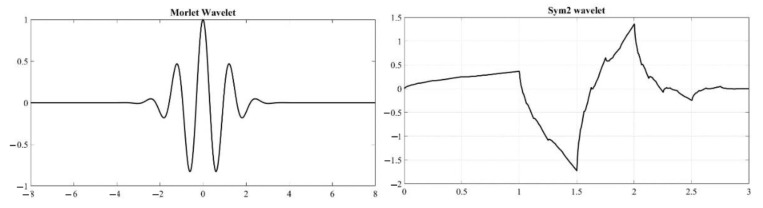
Wavelet function of Morlet and Sym2 wavelet.

It generates coefficients at various levels, and due to down sampling by 2, the resulting coefficient vector will be half of the total sample provided by the signal. DWT facilitates the decomposition of signals, leading to the identification of the important features of signals. In DWT, the maximum overlap discrete wavelet transforms (MODWT) method modifies only the filter and not the signal between levels, which means it can handle signals from any number of samples and need not necessarily be 2N. Since there is no down sampling, it will generate N coefficients. In this study, sym2 was used for MODWT analysis and Morlet wavelet was used for CWT analysis (refer to Figure 6). Morlet wavelets provide excellent time and frequency resolution [25]. It can capture the short burst of repeating cycles in acoustic signals to produce the best continuous wavelet transform of AE signals acquired. MODWT requires an orthogonal wavelet, such as Daubechies (db) or symlet (sym). This study used a second-order symlet, a modification of the db wavelet family. Sym 2 wavelet has a short filter length; hence, it can perform remarkably well for AE signal decomposition. 

As the sampling frequency of AE acquisition system was kept at 0.1 µs, it generated 3000 samples in 300 µs time. The sample domain represents time domain as number of samples. The MODWT also generated 3000 coefficients for each level. Seven decomposition levels were selected, thus generating seven detailed coefficients and one approximate coefficient. To properly analyse the effects of load on AE wave parameters, the relative energy of each level for three sample domains, i.e., 1 to 1000, 1001 to 2000, and 2001 to 3000 was generated for each sensor. As the sampling frequency was kept as 0.1 µs, the three sample domains represented the timings from 0 to 100 µs, 101 to 200 µs, and 201–300 µs. Total energy is then calculated by summing up all the coefficients (Equation (3)). Relative energy at each level of decomposition and sample domain is then calculated by taking ratio of respective energy to the total energy (Equation (4)).

(3) $$\mathrm{Total}\;\mathrm{energy}\;E=\overset{8}{\underset{i=1}{\sum }}\overset{3000}{\underset{j=1}{\sum }}w_{i,j}{}^{2}$$

where w_i,j_ denotes wavelet coefficient at i^th^ level and j^th^ sample while relative energy will be provided as

(4) $${\mathrm{Relative}\;\mathrm{energy}\;R}_{i,a}=\left(\overset{n}{\underset{a=m}{\sum }}w_{i,a}{}^{2}/E\right)\times 100$$

where w_i,j_ denotes wavelet coefficient at i^th^ level and a^th^ sample; m denotes starting sample number.

Analysis of the AE signal is necessary to understand the effect of stress conditions on waveform parameters. This can correlate with the severity of the damage, the load conditions, and the AE waveforms. This study used FFT and CWT to determine the frequency content of a genuine AE signal generated by a crack opening in a rail under load. On the other hand, DWT analysis reveals the shift in energy concertation with respect to frequency. The developed method can facilitate the separation of genuine signals, a robust denoising algorithm, and the identification of damage in rail health monitoring.

## 4. Results

### 4.1. Experimental Results of the Bending Test of Rail

**Figure 7:**
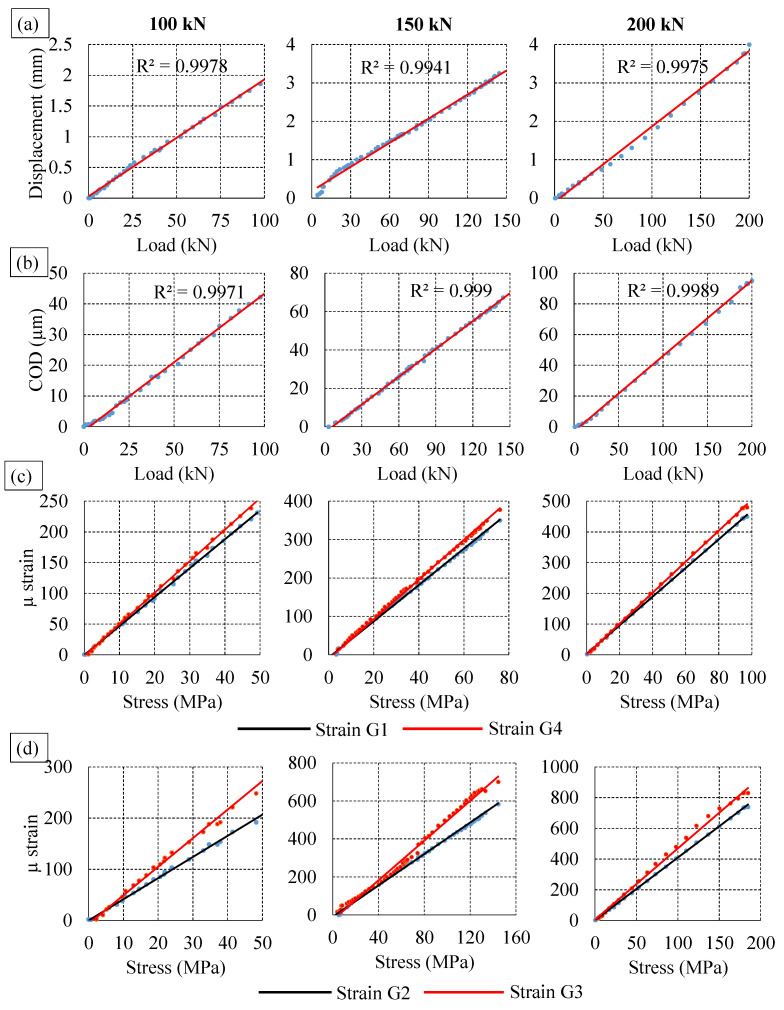
Experiment results (**a**) Vertical displacement (**b**) Crack opening displacement (**c**) Strain at far strain gauge (**d**) Strain at near strain gauge.

**Figure 8:**
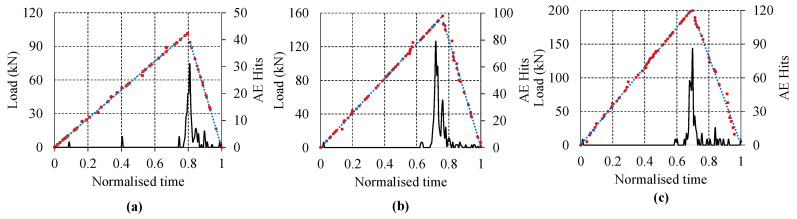
Recorded AE hits and load with respect to normalised time. (**a**) 100 kN, (**b**) 150 kN (**c**) 200 kN.

Bending test on rail was done on limited loads to avoid any instability problem on inverted rail. Load values were chosen in such a way that stresses remained the in elastic region. Figure 7 shows the results from the experiment with respect to loads. Vertical displacement and crack opening displacements are linear for all loads. Linear trend is also observed with bending strain and stress at the strain gauges near and far from the cracks. Strain gauges SG1 and SG4 are placed far from the crack on both sides symmetrically and both strain gauges have similar values. On the other hand, strain gauges SG2 and SG3 are placed near the cracks and have similar trend but indicate difference in values due to the presence of crack in the vicinity. The equipment used for AE data recording and load observations are different having different timing. Therefore, to compare loading and AE hits, time axis was normalised using the maximum recorded time during experiment. Figure 8 shows the number of AE hits and load corresponding to the normalised time of experiment for various loads. During the experiment, no significant AE hits were observed up to the load of 80 kN. An increasing trend of number of AE hits were observed with the increase in the magnitude of load. Highest number of hits can be observed at peak load in all cases. Initially, few AE hits were observed resulting from energy release due to impact of load adjustment. For all test, the number of waveforms recorded varies between 1200 to 2000. The number of AE waveform recorded are low due to proper AE waveform definition and high threshold values. This helps in eliminating lots of sources of noise generated from nearby environment. Sources of noise includes noise from loading machine, friction between contact surfaces, electrical source, and various other source. 

### 4.2. Clustering of AE Signals Generated during the Experiment

The clustering techniques outlined in the preceding section was applied using selected wave features in this experiment. Clustering is performed with various number of clusters and quality criteria is checked for finding out the optimal number of clusters. The silhouette score is a frequently used criterion for cluster quality; it returns a higher number to signify a better solution. To calculate the Silhouette score for each observation/data point, the distances between each observation belonging to each cluster must be determined.

The average distance between an observation and all other data points in a cluster. This distance is referred to as the mean intra-cluster distance, denoted by a.

The average distance between the observation and the nearest cluster’s other data points. This distance is also known as the mean distance to the nearest cluster, denoted by b.

Silhouette score, S for each sample is calculated by Equation (5):

(5) $$S=\frac{\left(b-a\right)}{\max\left(a,b\right)}$$

**Figure 9:**
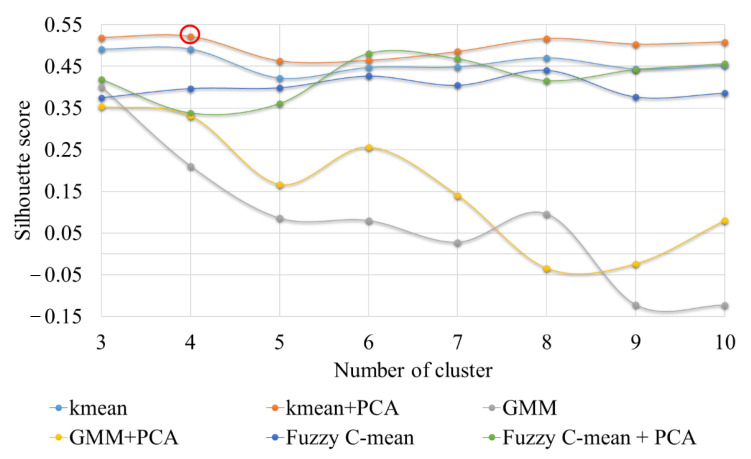
Silhouette score for each algorithm with respect to number of clusters.

**Figure 10:**
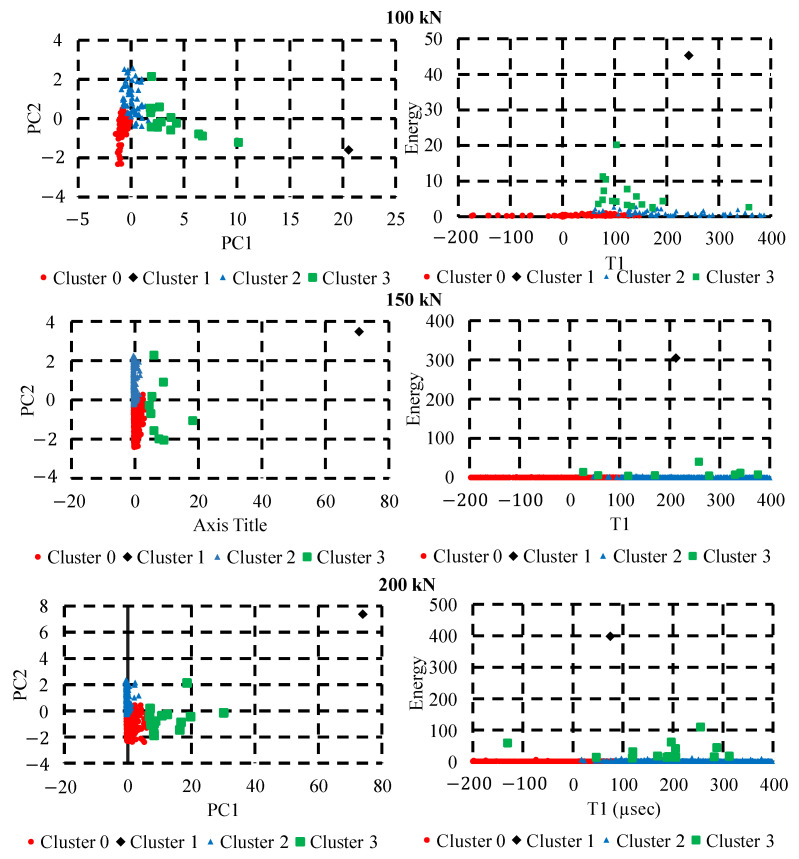
Clustering results for all three experiments; Cluster with respect to principal components (left side image); Cluster with respect to energy and rise time(right side image).

**Figure 11:**
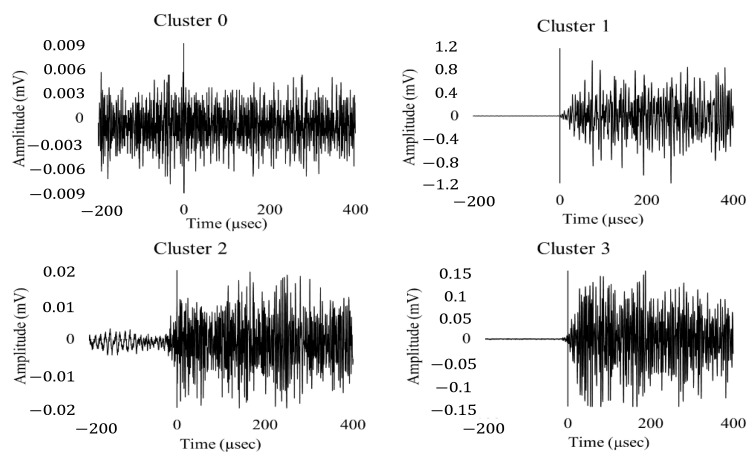
Sample signal from each cluster from AE dataset generated under 150 kN load.

**Figure 12:**
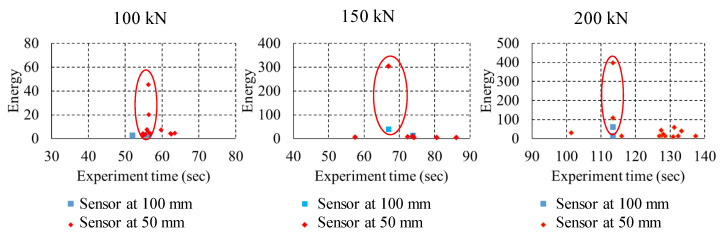
Experiment time vs. energy for AE signals in cluster 1 and 3.

Silhouette score of each method with features and principal components are compared to observe best number of clusters and method. Based on Figure 9, it can be noticed that four clusters are optimal and that PCA combined with k-means yields highest silhouette score as compared to all algorithms. Four distinct clustering was produced using k-means with PCA inputs. Figure 10 (left side image) shows the clusters with respect to the principal components 1 and 2. Both principal components together provide 97% variation in features hence helpful in reducing dimension without having much loss in information. As the principal components do not have any physical meaning, the cluster numbers obtained are then assigned with each AE signal. The right-hand image of Figure 10 shows the clusters with respect to energy and rise time. It can be easily observed for all three loading conditions, cluster 0 and cluster 2 are noisy datasets and there may not contain any usable AE signals as both signals are having very low energy components. On the other hand, two distinct clusters with high energy signals are also observed which can be contain actual signals. The sample signal from each cluster can be observed in Figure 11. The signals belonging to cluster 1 and 3 are separated out from the dataset and plotted against their time of occurrence during the experiment. The peak energy signals for both channels can be observed indicating energy release during the peak load (Refer Figure 12).

### 4.3. Analysis of Peak Energy Acoustic Signals

**Figure 13:**
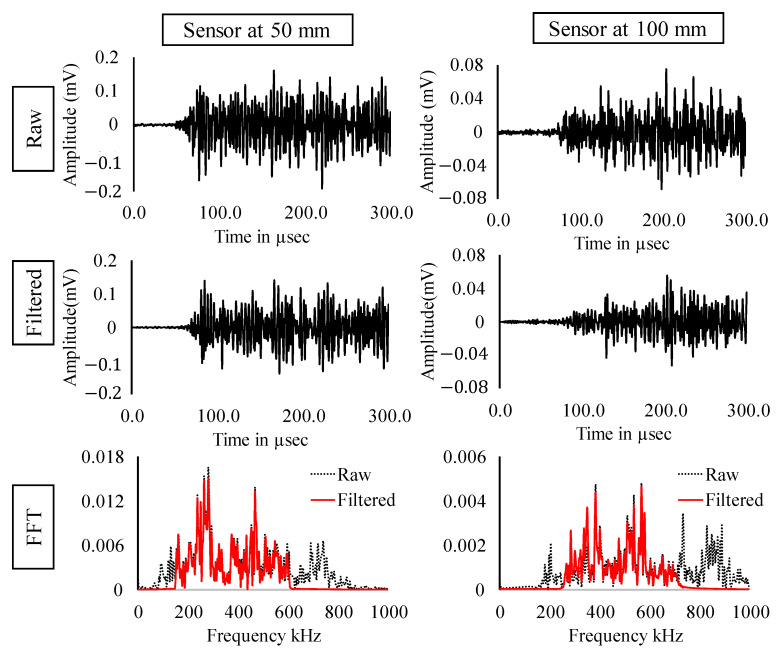
Raw, filtered and FFT of AE signals occurring at load = 100 kN.

**Figure 14:**
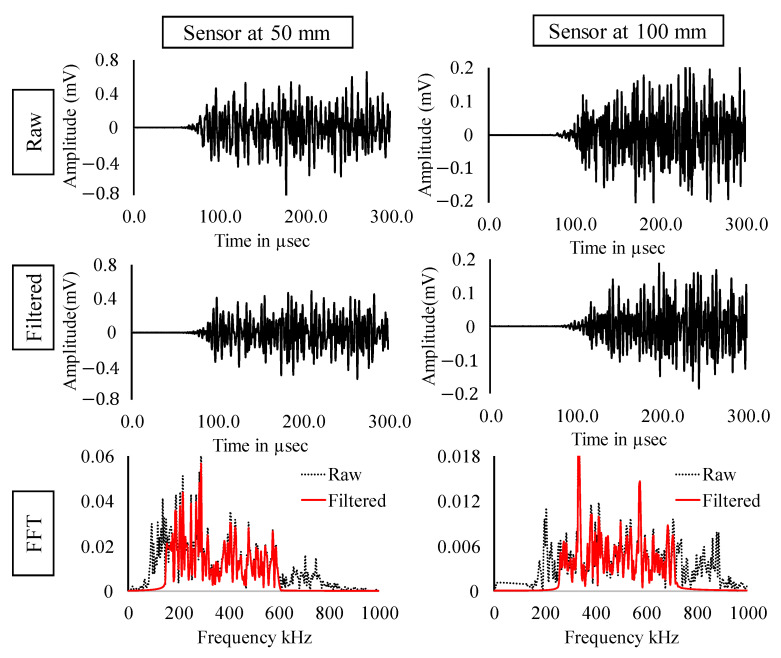
Raw, filtered and FFT of AE signals occurring at load = 150 kN.

**Figure 15:**
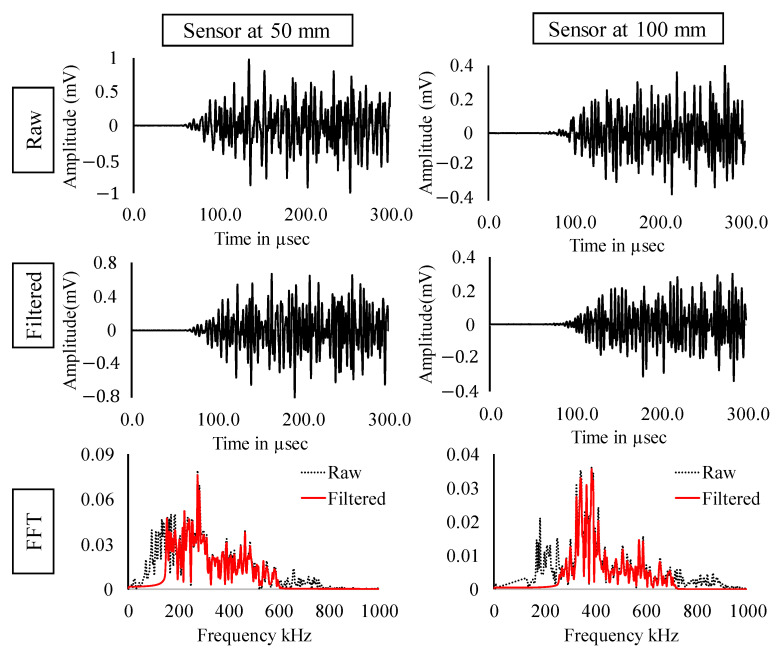
Raw, filtered and FFT of AE signals occurring at load = 200 kN.

In the previous section, the experimental results and the results from clustering were discussed. In this section, signals occurring at maximum loads are considered (Refer Figure 12). Figure 13, Figure 14 and Figure 15 show the raw, filtered, and fast Fourier transform (FFT) of AE signals occurring at loads 100 kN, 150 kN and 200 kN, respectively. The amplitude of signals shows an increasing trend indicating higher energy release with respect to increasing loads. Significant loss of energy can also be noticed in the signal from the sensor at 100 mm as compared to the sensor at 50 mm. Due to the complex boundaries of the rail section, the AE wave can be observed to have higher dispersion. Peak frequency and major energy in frequency domain can be observed in all the three cases in between 140 to 400 kHz, indicating the distinctive property of AE wave generated due to crack opening.

**Figure 16:**
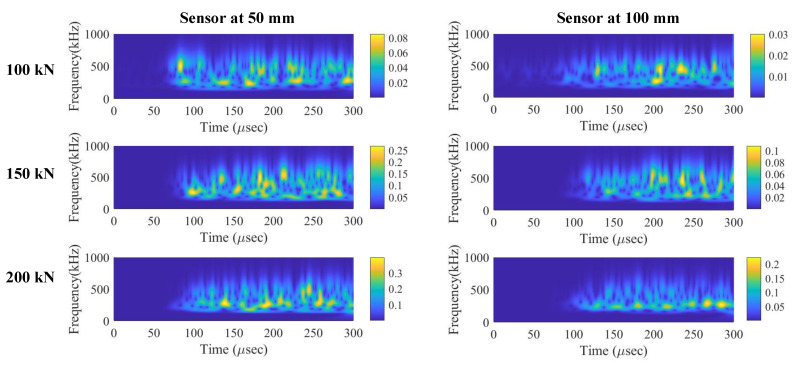
Continuous wavelets transform of AE signals at both sensors.

**Figure 17:**
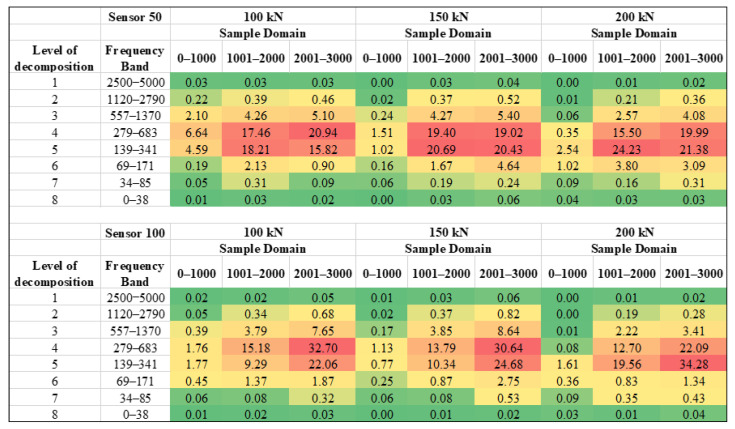
Comparison of relative energy generated through MODWT of signals for various loads. [Colour gradient: red to green corresponds to high to low relative energy].

Continuous wavelet transform represents the signal in time and frequency domains. Figure 16 shows the two-dimensional view of scalogram for all the signals. It can be observed that the frequency content varies significantly with respect to the time due to dispersion. Thus, the change in coefficient with respect to time and frequency may not be noticed accurately in scalogram. Nevertheless, discrete wavelet transform of signal provides considerably more information. Figure 17 represents the relative energy calculated at various levels of decomposition and three sample domains.

**Figure 18:**
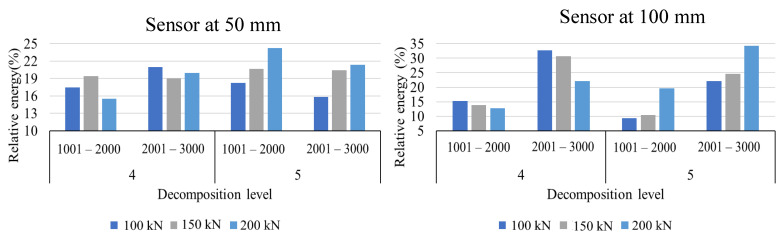
Change in waveform parameters with respect to load (a) Shift in relative energy for 4 and 5 decomposition levels with respect to sample domain.

It is evident that the frequency levels 4 and 5 is predominantly present in all the signals. The sensor closer to the crack has concentrated energies in these two levels, while the sensor far away from the crack has more distribution of energy. Figure 18 shows the variation of relative energy for levels 4,5, and sample domains 1001–2000 and 2001–3000. For both the sensors, the relative energy in decomposition level 5 is increasing as compared to level 4, with increase in load. The maximum amplitude of AE signals increases due to an increase in load-induced energy release from the rail section’s crack. The energy of a signal is directly proportional to the square of frequency and amplitude. Consequently, an inverse relationship between the frequency and amplitude of a signal. Therefore, an increase in load induces a relative energy shift from the higher frequency domain (279–683 kHz) to the lower frequency domain (139–341 kHz) with an increase in amplitude. Predictably, an increase in the severity of crack under stress may result in the discharge of signals with high amplitude and the high energy concentration in the relatively lower frequency domain.

**Figure 19:**
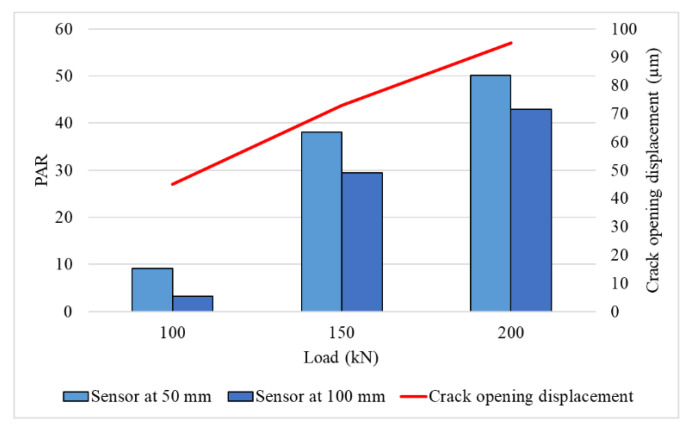
Variation of PAR and crack opening displacement with respect to load.

Apart from the frequency shift, there is the effect of loading on the amplitude of signals. It can be observed by taking the ratio between peak amplitude of actual signal and the average of peak amplitude of noisy dataset (i.e., signals in cluster 0 and cluster 2). This ratio is defined as peak amplitude ratio (PAR) as shown in Equation (6). Figure 19 shows the variation in PAR and crack opening displacement with respect to load. A linear relationship can be observed with load, COD, and PAR. In practice, stress concentration and crack opening depend on the extent of damage and applied load. Hence, analysing the relative amplitude of signals can provide insights on damage.

(6) $$\mathrm{Peak}\;\mathrm{amplitude}\;\mathrm{ratio}\;\left(\mathrm{PAR}\right)=\frac{\mathrm{Peak}\;\mathrm{amplitude}\;\mathrm{of}\;\mathrm{main}\;\mathrm{signal}}{\mathrm{Average}\;\mathrm{of}\;\mathrm{peak}\;\mathrm{amplitude}\;\mathrm{of}\;\mathrm{all}\;\mathrm{signals}\;\mathrm{in}\;\mathrm{noisy}\;\mathrm{data}\;\mathrm{set}}$$

## 5. Conclusions

The objective of this article is to conduct experimental study on a rail section with transverse damage in order to properly understand the various waveform parameters of an AE wave. To replicate the crack opening in the rail section, a bending test on an inverted rail with 38% head damage was done. To begin with, clustering methods was utilised to identify the actual signals in the noisy dataset. Following that, a thorough investigation was conducted using the Fourier transform, continuous wavelet transforms, and discrete wavelet transforms. The crack opening displacement and acoustic emission signal measurements were analysed, and the following conclusions were drawn:

- Using a wave-based feature in the clustering method, four distinct clusters were produced from which low-energy clusters could be removed. This method accelerates the separation of required AE signals and results in a reduction of manual labour required.
- From analysis of the AE signal, it was discovered that the signals created by the fracture had a specific frequency content ranging between 140 kHz and 400 kHz.
- The discrete wavelet transforms of AE signals indicated an energy shift in each frequency band and sample region. With increasing load, a considerable shift in the energy distribution in the frequency bands 139–341 kHz and 279–683 kHz was detected.
- Additionally, the peak amplitude ratio was observed to be linearly related to the loads and crack opening displacement.

The performed experiment and analysis contribute towards the frequency and amplitude-based techniques for damage detection in rail using AE testing. Very few studies have been conducted to correlate various waveform parameters with damage in the rail section. Our study utilised signal processing techniques to obtain valuable insights into the behaviour of AE signals generated due to damage in the rail section. The study can be expanded to build more robust denoising and clustering algorithms based on energy and frequency-related findings. Additionally, further experiments and numerical simulation may be performed to understand better the wave characteristics and their variation depending upon the extent of the damage. Supervised and unsupervised machine learning techniques in complex non-destructive evaluation can be employed to do data analysis in a fast, reliable, and efficient manner.

## Figures and Tables

**Table 1:** Features for the clustering algorithm.

S. No.	AE Waveform Feature	Expression
1.	Energy	$E=\overset{N}{\underset{n=1}{\sum }}Y^{2}\left(n\right)$
2.	Root mean square of signal	$\mathrm{RMS}=\sqrt{\frac{\sum_{n=1}^{N}Y{\left(n\right)}^{2}}{N}}$
3.	Fast Fourier Transform Peak amplitude	$F\left(j\omega \right)=\overset{N-1}{\underset{k=0}{\sum }}f\left[k\right]e^{-j\omega \mathrm{kT}}$
4	Area under FFT	
5.	Peak frequency	
6.	Rise time	T1 = Time in microsecond to peak amplitude
7.	Skewness	$\frac{3\;*\;\left(\mathrm{Mean}\;-\;\mathrm{Median}\right)}{\mathrm{Standard}\;\mathrm{deviation}}$
8.	Kurtosis	$\frac{\mathrm{Fourth}\;\mathrm{central}\;\mathrm{moment}}{{\mathrm{Standard}\;\mathrm{deviation}}^{4}}$

**Table 2:** Filter design parameter.

Parameter	Value
Response Type	IIR
Design Method	Elliptic
Exactly match	Passband
Filter order	4
Sampling frequency	10,000 kHz
First stopband frequency	125 kHz
First passband frequency	150 kHz
Second passband frequency	600 kHz
Second stopband frequency	650 kHz

## Data Availability

Not applicable.

## References

[1] Bruzelius K., Mba D. (2004). An Initial Investigation on the Potential Applicability of Acoustic Emission to Rail Track Fault Detection. Ndt E Int..

[2] Bollas K., Papasalouros D., Kourousis D., Anastasopoulos A. (2010). Acoustic Emission Inspection of Rail Wheels. J. Acoust. Emiss..

[3] Hao Q., Wang Y., Shen Y., Zhang X. De-Noising of Rail Crack AE Signal Based on Wavelet Modulus Maxima. Proceedings of the 2015 IEEE International Instrumentation and Measurement Technology Conference (I2MTC).

[4] Liang B., Iwnicki S., Ball A.E., Young A. (2015). Adaptive noise cancelling and time–frequency techniques for rail surface defect detection. Mech. Syst. Signal Process..

[5] Li D., Kuang K.S.C., Koh C.G. (2017). Fatigue crack sizing in rail steel using crack closure-induced acoustic emission waves. Meas. Sci. Technol..

[6] Pomponi E., Vinogradov A. (2013). A real-time approach to acoustic emission clustering. Mech. Syst. Signal Process..

[7] Das A.K., Suthar D., Leung C.K. (2019). Machine learning based crack mode classification from unlabeled acoustic emission waveform features. Cem. Concr. Res..

[8] Tang J., Soua S., Mares C., Gan T.-H. (2017). A Pattern Recognition Approach to Acoustic Emission Data Originating from Fatigue of Wind Turbine Blades. Sensors.

[9] Pashmforoush F., Khamedi R., Fotouhi M., Hajikhani M., Ahmadi M. (2014). Damage Classification of Sandwich Composites Using Acoustic Emission Technique and k-means Genetic Algorithm. J. Nondestruct. Eval..

[10] Satour A., Montrésor S., Bentahar M., Boubenider F. (2020). Wavelet Based Clustering of Acoustic Emission Hits to Characterize Damage Mechanisms in Composites. J. Nondestruct. Eval..

[11] Van Steen C., Verstrynge E. (2022). Signal-Based Acoustic Emission Clustering for Differentiation of Damage Sources in Corroding Reinforced Concrete Beams. Appl. Sci..

[12] Li D., Wang Y., Yan W.-J., Ren W.-X. (2021). Acoustic emission wave classification for rail crack monitoring based on synchrosqueezed wavelet transform and multi-branch convolutional neural network. Struct. Health Monit..

[13] Mahajan H., Banerjee S. (2022). A machine learning framework for guided wave-based damage detection of rail head using surface-bonded piezo-electric wafer transducers. Mach. Learn. Appl..

[14] Noseda F., Marnet L.R., Carlim C., Costa L.R., Junior N.d.M., Calôba L.P., Soares S.D., Clarke T., Jacques R.C. (2021). A Neural Network System for Fault Prediction in Pipelines by Acoustic Emission Techniques. Res. Nondestruct. Eval..

[15] Garrett J.C., Mei H., Giurgiutiu V. (2022). An Artificial Intelligence Approach to Fatigue Crack Length Estimation from Acoustic Emission Waves in Thin Metallic Plates. Appl. Sci..

[16] Suwansin W., Phasukkit P. (2021). Deep Learning-Based Acoustic Emission Scheme for Nondestructive Localization of Cracks in Train Rails under a Load. Sensors.

[17] Palmer I.G., Brindley B.J., Harrison R.P. (1974). The relationship between acoustic emission and crack opening displacement measurements. Mater. Sci. Eng..

[18] (2006). Railway Applications-Track-Aluminothermic Welding of Rail-Part 1: Approval of Welding Processes.

[19] Li G., Zhao Z., Li Y., Li C.-Y., Lee C.-C. (2022). Preprocessing Acoustic Emission Signal of Broken Wires in Bridge Cables. Appl. Sci..

[20] MISTRAS Group, Inc. (2014). Express-8 AE System User’s Manual.

[21] Al-Jumaili S.K., Holford K., Eaton M., McCrory J., Pearson M., Pullin R. (2015). Classification of acoustic emission data from buckling test of carbon fibre panel using unsupervised clustering techniques. Struct. Health Monit..

[22] Farhidzadeh A., Dehghan-Niri E., Salamone S. Gaussian Mixture Modeling of Acoustic Emissions for Structural Health Monitoring of Reinforced Concrete Structures. Proceedings of the SPIE—The International Society for Optical Engineering.

[23] Saldarriaga-Zuluaga S.D., López-Lezama J.M., Muñoz-Galeano N. (2021). Optimal Coordination of Over-Current Relays in Microgrids Using Principal Component Analysis and K-Means. Appl. Sci..

[24] Abraham D.A., Thomas H., Bradley D. (2017). Chapter 11—Signal Processing. Applied Underwater Acoustics; Neighbors.

[25] Ciampa F., Meo M. (2010). Acoustic emission source localization and velocity determination of the fundamental mode A_0_ using wavelet analysis and a Newton-based optimization technique. Smart Mater. Struct..

